# Determination of Folic Acid Using Biosensors—A Short Review of Recent Progress

**DOI:** 10.3390/s21103360

**Published:** 2021-05-12

**Authors:** Alessio Di Tinno, Rocco Cancelliere, Laura Micheli

**Affiliations:** 1 Department of Chemical Sciences and Technologies, University of Rome Tor Vergata, Via della Ricerca Scientifica 1, 00133 Rome, Italy; alessio.ditinno@alumni.uniroma2.it (A.D.T.); rocco.cancelliere@uniroma2.it (R.C.); 2 Department of Electrical and Information Engineering, University of Cassino and Southern Lazio, Via Gaetano di Biaio 1, 03043 Cassino, Italy; 3 CNR—National Research Council of Italy, Institute of Crystallography (IC), Via Salaria Km 29,300, 00015 Rome, Italy; 4 INBB—Consorzio Interuniversitario Istituto Nazionale di Biostrutture e Biosistemi, Viale Medaglie d’Oro 305, 00136 Rome, Italy

**Keywords:** folic acid, real samples, analytical methods, electrochemical tools

## Abstract

Folic acid (FA) is the synthetic surrogate of the essential B vitamin folate, alternatively named folacin, pteroylglutamic acid or vitamin B_9_. FA is an electroactive compound that helps our body to create and keep our cells healthy: it acts as the main character in a variety of synthetic biological reactions such as the synthesis of purines, pyrimidine (thus being indirectly implied in DNA synthesis), fixing and methylation of DNA. Therefore, physiological folate deficiency may be responsible for severe degenerative conditions, including neural tube defects in developing embryos and megaloblastic anaemia at any age. Moreover, being a water-soluble molecule, it is constantly lost and has to be reintegrated daily; for this reason, FA supplements and food fortification are, nowadays, extremely diffused and well-established practices. Consequently, accurate, reliable and precise analytical techniques are needed to exactly determine FA concentration in various media. Thus, the aim of this review is to report on research papers of the past 5 years (2016–2020) dealing with rapid and low-cost electrochemical determination of FA in food or biological fluid samples.

## 1. Introduction

Folic acid (FA) or pteroylglutamic acid is a water-soluble B-complex vitamin and, due to its extremely important functions, represents an essential constituent of the human diet. It is the synthetic substitute of the essential B vitamin folate, also known as pteroylglutamic acid, folacin or vitamin B9. The IUPAC name of FA is (2S)-2-[[4-[(2-amino-4-oxo-1H-pteridin-6-yl)methylamino]benzoyl]amino]pentanedioic acid.

**Figure 1:**
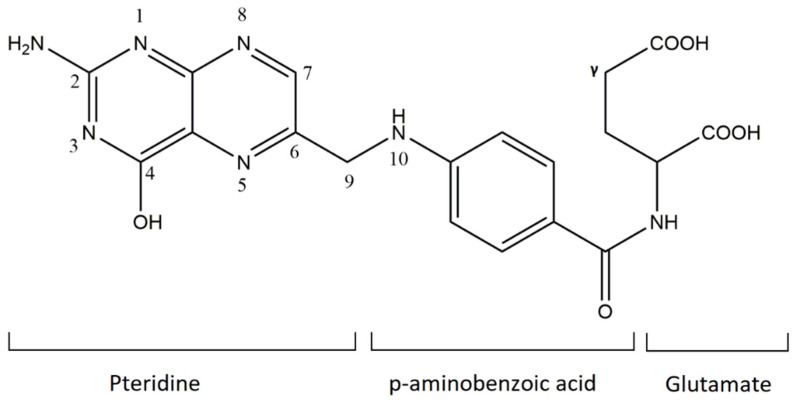
Schematic representation of folic acid (mono-glutamate derivate).

Beginning in the 2000s, FA and folate derivatives received increasing interest due to their importance for human well-being and due to growing understanding of the consequences of deficiency [1,2]. These molecules are considered essential compounds, because precursors of fundamental coenzymes are needed in many crucial biochemical reactions. FA molecular structure is made up of three components: a pteridine portion linked by through p-aminobenzoic acid to L-glutamic acid (Figure 1). The acyl group coming from the pteroic acid is a pteroyl group [3].

FA, though, has no biological activity itself but acts as fundamental precursor of a group of crucial coenzymes. Furthermore, FA is not produced by human body, and thus it must be obtained through diet (in the folate form), including liver, yolk, kidney beans, green leafy vegetables and fresh fruits [4]. Due the extreme importance of FA in cell proliferation, its assimilation is more and more frequently achieved by taking food supplements [5]. Indeed, all cells need folate in its reduced form in order to renew their cellular components. Tetrahydrofolate, for instance, acts as a cofactor in some essential metabolic pathways, such as DNA synthesis and biological methylation [6]. Different organisms exploit different strategies to obtain folate: plants and some microorganisms can produce folate from scratch with minimal variations of the same biosynthetic pathway [7,8,9]. Conversely, mammals, being auxotrophs, obtain folate through their diet or by exploiting intestinal bacteria capable of synthesizing it [10,11]. Despite all the different functions of the folate coenzyme, the main one is to transfer one-carbon groups in a variety of synthetic reactions. This capability varies depending upon the state of oxidation of the transferred group. Furthermore, FA can participate in a plethora of key reactions for fundamental cell functions: among the most important reported are the synthesis of purines, pyrimidines (and thus, indirectly, in the synthesis of DNA) and methionine, and the repair and methylation of DNA. Folate deficiency may result in degenerative conditions, such as neural tube defects in developing embryos and megaloblastic anaemia at any age [12]. Being water-soluble, folate cannot be stored in the human body and consequently is continuously lost. Therefore, its deficiency is one of the most commonly found vitamin deficits. The effective folate lack in the world is not well understood even though it appears a usual condition for many vulnerable classes. The use of so-called FA antagonists in certain disease (such as cancer [13], leukaemia [14], psoriasis [15], rheumatoid arthritis [16,17], polymyositis [18,19], dermatomyositis [20,21] and so on) may seem to suggest that a folate surplus in the diet would be harmful. Moreover, there have recently been rising concerns that FA supplementation could actually increase the risk of cancer frequency [22], as animal and human studies have indicated that high folate status may promote the progression of preneoplastic and undiagnosed neoplastic lesions [23,24]. There is little evidence to support such a view, nor it is well understood if FA supplements hamper the therapeutic effectiveness of these medications. Due to FA’s ability to itself act as dihydrofolate reductase inhibitor, it could quite possibly be not only reliable but even advantageous in the treatment of these disorders [25,26]. For this reason, FA supplements are widely used to prevent and handle folate deficiency in at-risk groups and also to prevent adverse events associated with antifolate medications [27]. A daily intake of FA, through the commercially available supplements, is recommended to fertile and pregnant women in order to limit possible neural disorders in developing fetuses [28]. Folate is highly recommended also in subjects with heart failure due to its ability to lower the blood-homocysteine level, which has been linked to increased risk of cardiovascular events [29,30]. Moreover, FA promotes the formation of vigorous and healthy red blood cells [31,32]. However, some countries decided to not adopt FA fortification, being afraid of the possible negative consequences [33,34]. All these different benefits and disadvantages have led the research world to develop and optimize analytical methods, which can dependably and accurately monitor the FA concentration in natural sources, fortified foods, and multivitamin dietary supplements. In Europe, the EFSA (European Food Safety Authority) balances the fortification of flour (wheat and maize), establishing a minimum and maximum of 140 and 220 μg of FA per 100 g. For women with a history of congenital malformation, the recommended daily dose is 5 mg to reduce the risk of recurrence of the problem. A plethora of different analytical methods have been applied to determine FA in natural sources, using laboratory instrumentation as thermogravimetry [35], spectrophotometry [36,37], high performance liquid chromatography (HPLC) [38,39] with mass spectroscopy [40,41], colorimetry [42,43], fluorescence [44], electrophoresis [45,46] and so on (Table 1). Recently, an interesting overview about the future trends in the market for electrochemical biosensing has been proposed in [47], in which the current outline of the sensors and biosensors market is summarized. Some of the most recent advances are discussed, along with future prospects for biosensing development that could make an impact on the future global market. This short review reports on research papers of the past 5 years (2016–2020) dealing with the electrochemical determination of FA in food or biological fluid samples.

## 2. Electrochemical Determination

In recent decades, the growing interest in FA, due to its physiological importance, has involved the development of a plethora of methods for its determination [49]. Thus, a sensitive, specific and easy-to-use way to quantify FA is crucial. In this section, a collection of the most relevant and original electrochemical sensors of the past 5 years for FA determination is reported. In particular, two different subsections are mentioned: traditional and screen-printed based sensors.

### 2.1. Traditional Sensors

**Figure 2:**
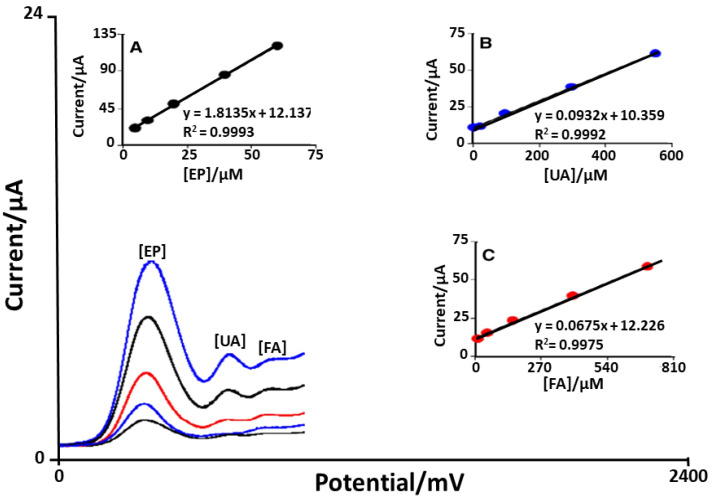
SWVs of 2FTNEMCNPPE in 0.1 M PBS (pH 7.0) with various concentrations (μmol L^−1^) of EP, UA FA in a mixed solution: (1) 5.0 + 5.0 + 10.0, (2) 50.0 + 25.0 + 10.0, (3) 150.0 + 100.0 + 20.0, (4) 400.0 + 300.0 + 40.0, (5) 700.0 + 550.0 + 60.0. (**A**–**C**): plots of peak currents as a function of EP, UA and FA concentration, respectively [50].

In 2018, Mohammadi et al. [50] aimed to fabricate an FA-sensitive platform based on manganese ferrite nanoparticles modified by means of trimethoxy silane (3-amynopropyl). In particular, in this study, core shell magnetic nanoparticles (CMNP) were involved in order to produce 2FTNE ((2-(4-Ferrocenyl-[1,2,3]triazol-1-yl)-1-(naphthalen-2-yl) ethanone), successively used in the modification of CMPE paste electrodes (2FTNE-modified CMNP paste electrodes), whereby this platform has been carefully characterized. The main goal of this research study was the concurrent measurements of epinephrine (EP), uric acid (UA) and FA. For this purpose, the concentrations of these analytes were simultaneously changed over time. The analysis was conducted using 2FTNE-modified CMNP paste electrodes (2FTNEMCPPE) and square wave voltammetry (SWV) as electrochemical technique, as reported in Figure 2. It is possible to observe that three well-distinguished anodic peaks at defined potentials of 430, 730 and 930 mV, corresponding to the oxidation peaks of EP, UA and FA, respectively, were obtained, which confirmed that the concurrent measurements of these analytes was possible (Figure 2). With the use of bare CPE, an overlapping voltammogram for the analytes was obtained. The sensitivity of the 2FTNEMCNPPE toward EP was 1.813 μA μmol^−1^ L, while without UA and FA, it was found to be 1.839 μA μmol^−1^ L. As a consequence, it is possible to have the independent or the concurrent measurements and quantification of EP, UA and FA. Finally, 2FTNEMCNPPE was applied to measure EP, UA and FA in an EP ampoule, an FA tablet and urine samples. All the different outcomes are summarized in Table 2. In addition, the recovery of EP, UA and FA of the samples spiked with known amounts of EP, UA and FA was assessed.

**Figure 3:**
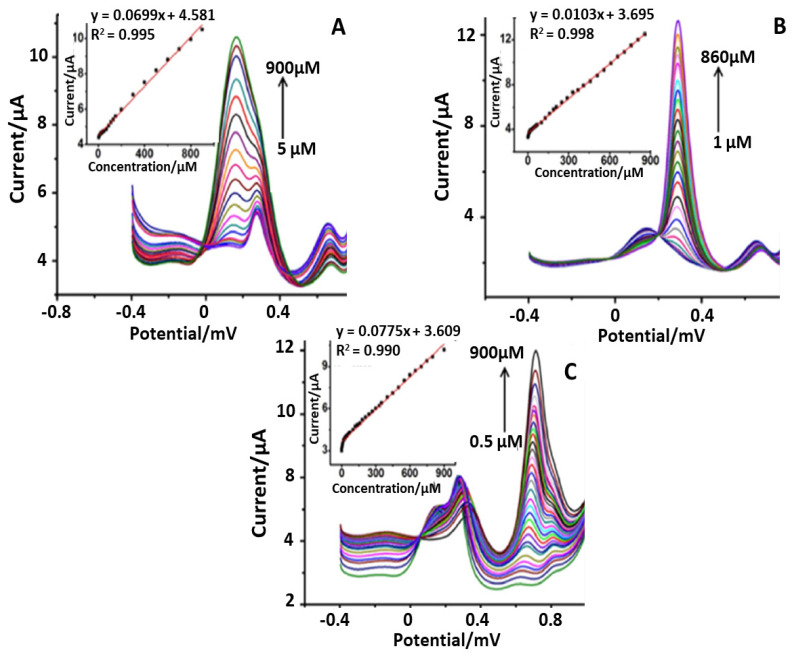
SWVs obtained for various concentrations of UA (5 to 500 μM) (**A**) and FA (1 to 800 μM) (**B**) at Mn SnO_2_/GCE in the presence of 200 μM AA in 0.1 M PBS (pH 6.0) and inreal samples (**C**). (insert of **A**–**C**): plots of the oxidation peak currents as a function of various concentrations of UA and FA, respectively [51].

Back in 2016, Lavanya et al. [51] developed a selective electrochemical sensor based on Mn doped SnO_2_ nanoparticles (NPs) modified glassy carbon electrode (Mn-SnO_2_/GCE). In particular, this platform was employed for the simultaneous determination of ascorbic acid (AA), uric acid (UA) and FA. These nanoparticles were synthesized by microwave irradiation and fully characterized using different spectroscopical and morphological techniques: X-ray diffraction (XRD), transmission electron microscopy (TEM), X-ray photoelectron spectroscopy (XPS), vibrating sample magnetometer (VSM), EIS, CV and SWV, respectively. AA is an important interfering analyte, which coexists with FA and UA in real body fluids. For this reason, the concentrations of UA and FA were determined in presence of a very high concentration of AA. The SWVs, reported in Figure 3, were obtained from the mixture of UA and FA in the presence of 200 µM AA.

It can be easily noticed (Figure 3A) that simultaneously increasing the FA and UA concentrations a corresponding linear increase in the anodic peak currents have been obtained. Thus, a linear relationship between analytes concentration and their anodic peak currents is established. Furthermore, using different concentrations of UA and FA (in the range 5.0 to 500 µM for UA and 1.0 to 500 µM for FA), detection limits of 0.025 and 0.038 µM, respectively, have been obtained. To verify the applicability of this platform (Mn-SnO_2_/GCE sensor) to real samples, AA and FA have been determined in pharmaceutical products. A recovery value corresponding to 99.4% (labeled content of FA mg/tablet 5, observed content of FA mg/tablet 4.88) was found using 3 wt% Mn-SnO_2_/GCE. Thus, the method studied could be reliably applied for the determination of FA and AA in commercial samples.

**Figure 4:**
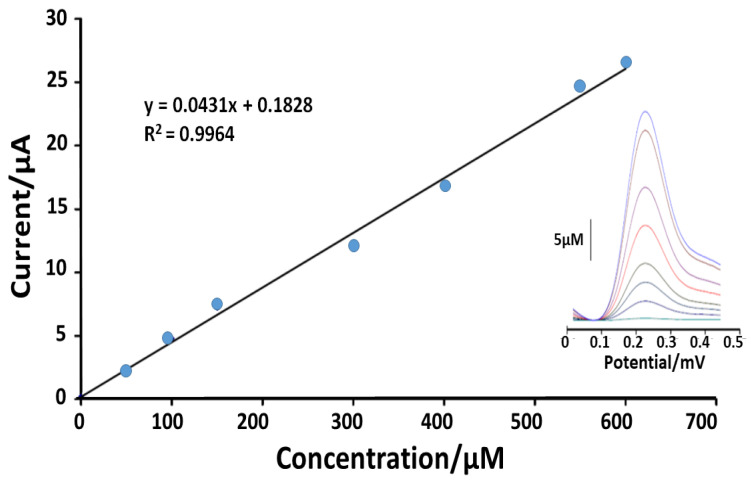
Current-concentration curve for the oxidation of FA in the range of 3.0 nM–250 µM. Inset: relative DP voltammograms of FA at surface of PE/CuO-CNTs/BDHFP [52].

Another important work, published in 2020 by Sadeghi et al. [52], proposed an electrochemical amplified FA sensor based on paste electrode (PE) modified with CuO-CNTs and 1-butyl-2,3-dimethylimidazolium hexafluorophosphate (BDHFP). Specifically, this device is based on the FA oxidation current registered by means of DP voltammograms, which increased 2.8 times using PE/M/CuO-CNTs-BDHFP with respect to PE. The modification produced an increase in the active surface area of PE (from 0.11 cm^2^ to 0.18 cm^2^ after modification with CuO-CNTs and BDHFP). DP voltammograms of different concentrations of FA were recorded on the surface of PE/M/CuO-CNTs/BDHFP with a linear dynamic range between 3.0 nM and 250 µM with a detection limit of 0.8 nM using PE/M/CuO-CNTs/BDHFP as an electrochemical sensor (Figure 4).

The ability of PE/CuO-CNTs/BDHFP was also tested in real samples. To this purpose, it is employed in the determination of FA in orange and apple juices by applying the standard addition method (data repeated in Table 3). The results, summarized in Table 3, confirmed the powerful performances of this sensors for real sample analysis.

**Figure 5:**
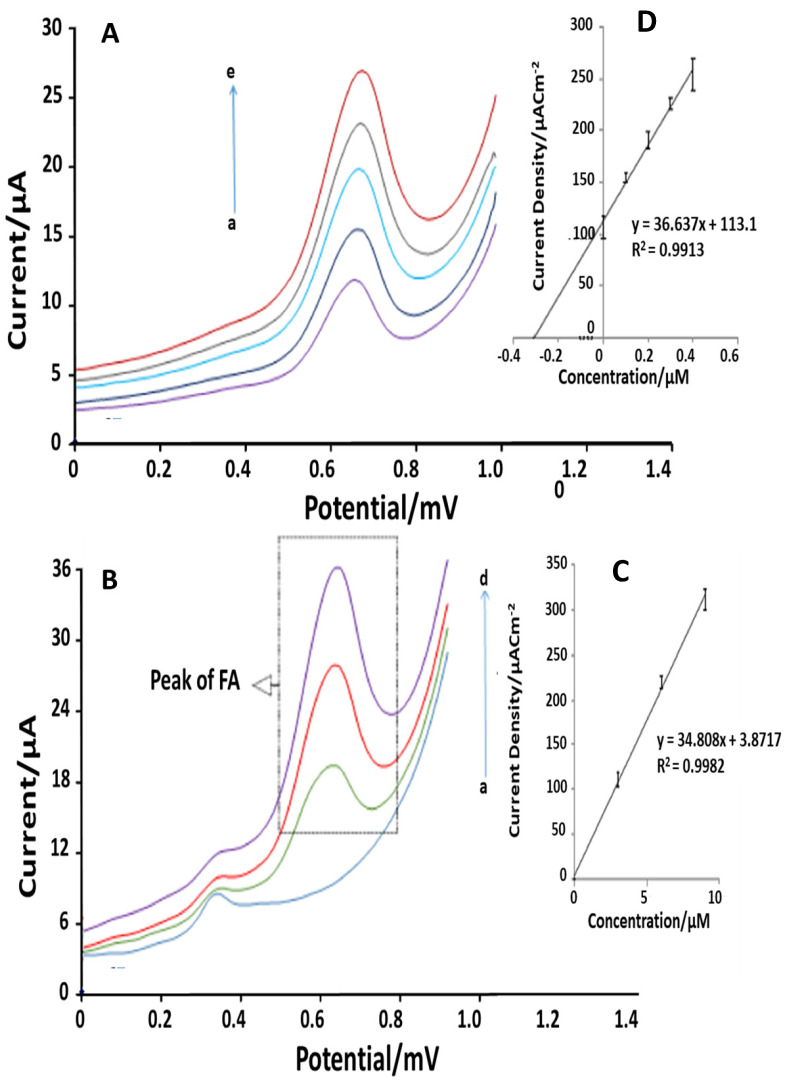
(**A**): Differential pulse voltammograms of CPE in 0.1 M PB (pH = 8.0) and drug tablet (3.0 µM FA) containing different concentrations of pure FA: a–e corresponds to 0.0–4.0 µM of pure FA. (**B**): Differential pulse voltammograms of CPE in 0.1 M PB (pH = 8.0) and 0.2 mL of a urine sample containing a different concentration of FA: the letters a–d correspond to 0.0–9.0 µM of FA. (**C**,**D**): Standard addition curve of the peak current density J (µM cm^−2^) (extrapolated from respectively voltammograms A and B) vs. the concentration of FA [53].

Mollaei et al. [53] in 2019 reported an electrochemical sensor for FA detection based on the adsorption of a cationic surfactant (n-dodecylpyridinium chloride, DPC), at the surface of carbon paste electrode (CPE). The electrochemical performances in FA detection were compared with cetyltrimethylammonium bromide (CTAB). This quantitative analysis is performed using different voltammetric techniques: the differential pulse voltammetry (DPV), cyclic voltammetry (CV) and chronocoulometry (CC). Moreover, the determination of FA in urine and pharmaceuticals were performed in order to demonstrate the capability of the improved method and its reproducibility in real sample matrix (Figure 5). To overcome the matrix effect drawback, in both cases, the conventional standard addition method was utilized. The results are summarized in Table 4, indicating acceptable recoveries (ranging between 94.1 and 103.5) and working range.

**Figure 6:**
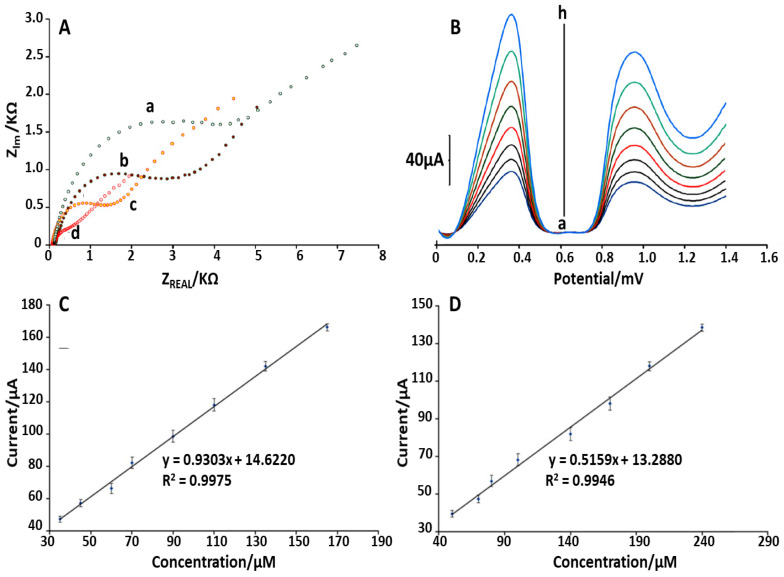
(**A**): Nyquist diagrams of (a) CPE, (b) FeNi_3_/rGO/CPE, (c) HMPF_6_/CPE and (d) FeNi_3_/rGO/HMPF_6_/CPE in 1.0 mM [Fe(CN)_6_]^3−,4−^. (**B**): SW voltammograms of solution containing TBHQ and FA at the FeNi_3_/rGO/HMPF_6_/CPE; (a) 35.0 + 50.0; (b) 45.0 + 70.0; (c) 60.0 + 80.0; (d) 70.0 + 100.0; (e) 90.0 + 140.0; (f) 110.0 + 170.0; (g) 135.0 + 200.0 and (h) 165.0 + 240.0 μM. (**C**): The Ipa vs. TBHQ concentration obtained from SW voltammograms. (**D**): The Ipa vs. folic acid concentration obtained from SW voltammograms [54].

Looking at Figure 5, it can be noticed that CPE in the presence of DPC has good operating properties such as selectivity, sensitivity, stability, repeatability, low detection limit (2.9 nM) and wide linear concentration range (0.01–10.69 mM). Finally, Tahernejad-Javazmi et al. [54] in 2019, using previous experience with electrochemical sensor employing CuO nanoparticle decorated on single wall carbon nanotubes (CuO/SWCNTs) nanocomposite and 1-butyl-3-methylimidazolium hexafluorophosphate [55], developed an electroanalytical sensor based on reduced graphene oxide (GO) modified carbon paste electrode (CPE). In particular, the GO modified platform were functionalized with FeNi_3_ (FeNi_3_)/rGO-ionic liquid n-hexyl-3-methylimidazolium hexafluoro phosphate (HMPF_6_). This platform was then employed for the simultaneous determination of FA and TBHQ (the antioxidant additive tertbutylhydroquinone), respectively. The modification process of CPE with FeNi_3_/rGO and HMPF_6_ was followed by EIS measurements (Figure 6A) and using 1.0 mM [Fe(CN)_6_]^3/,4−^ as an electrochemical probe. Using this technique and going from bare electrode to FeNi3-modifed platform (FeNi_3_rGO/HMPF_6_/CPE), an important decrease in the charge transfer resistance (Rct) value (from 6480 Ω to 870 Ω) was observed. This confirmed that the FeNi_3_/rGO and HMPF_6_ based modification improved conductivity and the electron transfer process at CPE’s interface. The FeNi_3_/rGO/HMPF_6_/CPE performances have been compared to previous published methods [56,57]. The results, displayed in Table 5, show the applicability of this modified CPE for real sample analysis.

### 2.2. Screen Printed Electrodes (SPEs)-Based Sensors

Screen printed electrodes (SPEs)-based sensors are an improvement of traditional sensors for performing rapid and accurate in situ analyses and for the development of portable devices. In the past 5 years, few biosensors based on SPEs were developed for the detection of FA in pharmaceutical and biological products, as summarized in Table 6.

The great results have been achieved in the application of SPEs for the detection of FA and the main results are described below in detail.

**Figure 7:**
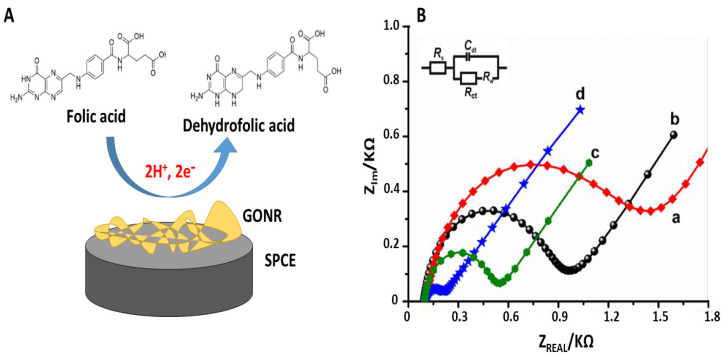
(**A**): Scheme of the electrochemical oxidation of FA on GONR/SPE. (**B**): EIS curves of unmodified SPE (a) MOS_2_-SPE (b), GNS-MoS_2_ /SPE (c), MoS_2_-AuNPs/SPE, (d) in 0.1 M KCl containing 5 mM Fe(CN)_6_ ^3−/4−^. Frequency: 0.1 Hz to 100 kHz [63].

In 2016, Mani et al. [62] developed a facile method to determine FA in real samples. In this work, the preparation of a ternary nanocomposite made of graphene nanosheets (GNS), molybdenum disulfide (MoS_2_) and gold nanoparticles (AuNPs) were detailed and its electrochemical sensing suitability studied. This ternary nanocomposite, GNS-MoS_2_-AuNPs, characterized by particle sizes of about 10–50 nm, is deposited onto the surface of screen-printed electrodes (SPE) and applied for the quantification of FA. Due to the good synergic effect between GNS, MoS_2_ and AuNPs, the composite shows excellent electrocatalytic ability. Impedance and electrochemical attributes of the nanocomposite were carefully studied: charge transfer resistance (R_ct_) compared (Figure 7) for unmodified SPE, MoS_2_/SPCE, GNS–MoS_2_/SPCE and GNS–MoS_2_–AuNPs/SPE. The R_ct_ values are reported in the following order: unmodified SPE > MoS_2_/SPE > GNS–MoS_2_/SPE > GNS–MoS_2_–AuNPs/SPE, respectively. In particular, R_ct_ obtained at GNS–MoS_2_–AuNPs/SPE is the lowest compared with control electrodes, indicating lowest resistance at this electrode interface. Thus, the electrochemical impedance spectroscopy (EIS) results revealed that the GNS–MoS_2_–AuNPs composite interface presents a better electrical conductivity than the other platforms. Furthermore, this EIS study was followed by a potentiometric investigation in amperometry. Using this technique, the determination of FA in human urine sample occurs in a wide linear range of 50 nM–1150 µM and displays low detection limit of 38.5 nM. To apply these platforms in real samples, a recovery study was conducted, obtaining satisfactory results (values range from 97.16 to 98.55%). The sensor performance of the GNS–MoS_2_–AuNPs is either superior or comparable to the previously reported electrodes [65,66,67].

Safaei and co-workers [63] in 2019 proposed a device for the simultaneous determination of FA and epinephrine (EP). This sensitive and convenient electrochemical sensor is based on Fe_3_O_4_@SiO_2_/GR nanocomposite modified graphite SPE using cyclic voltammetry as detection tools. In particular, cyclic voltammograms (CVs) were recorded in the presence of analytes after having cycled the potential 20 times at a scan rate of 50 mV s^–1^. The peak potentials were unchanged and the currents decreased by less than 2.3%. Therefore, at the surface of Fe_3_O_4_@SiO_2_/GR /SPE, not only the sensitivity increases, but the fouling effect of the analyte and its oxidation product also decreases. To ascertain the analytical applicability of the proposed method, real samples matrices were evaluated. In particular, the simultaneous determination of epinephrine and FA, using the standard addition method and DPV as analytical technique, in human blood serum, urine, epinephrine injection and folic acid tablets samples, were analyzed in depth. In Table 7, the relative results are reported.

Satisfactory recoveries were found for epinephrine and FA, as reported in the above table. Reproducibility is reported as mean relative standard deviations (RSD%). The sensor exhibited notable electrochemical activity towards the oxidation of epinephrine and FA, and solved the overlapping anodic peak outcomes of EP and FA into two well-defined peaks.

**Figure 8:**
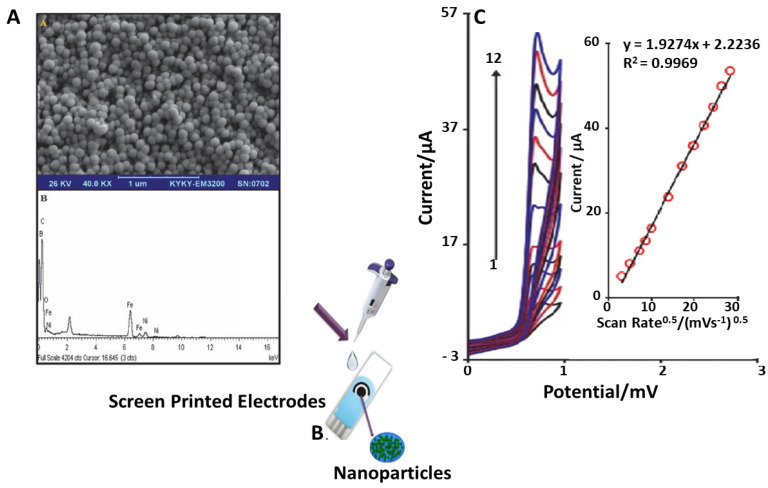
(**A**): SEM Micrograph of NiFe_2_O_4_ and its EDX. (**B**): deposition of NPs on SPE with drop casting. (**C**): CVs of NFO/SPE in 0.1 M PBS (pH 7) containing 150 µM of FA at various scan rates; numbers 1–12 correspond to 10, 25, 50, 75, 100, 200, 300, 400, 500, 600, 700 and 800 mV s^−1^, respectively. Inset: variation of anodic peak current vs. square root of scan rate [64].

Safaei et al. [64], in 2019, successfully synthesized and used NiFe_2_O_4_ (NFO) nanoparticles in order to develop a modified novel voltammetric sensor for determination of FA in urine samples. The morphological characterization of NFO nanoparticles was examined by means of scanning-electron microscopy (SEM), and it was observed that all the particles are nearly spherical, not agglomerated and less than 10 nm. In Figure 8C, cyclic voltammograms (CVs) are depicted, obtained using NFO-modified SPE. Analyzing equal concentration of substrate, NFO/SPE shows much higher anodic peak currents for the oxidation of folic acid compared to the unmodified SPE. This indicates that the modification of bare SPE with NiFe_2_O_4_ nanoparticles has significantly improved the performance in terms of electron transfer process between the electrode and the folic acid. In Figure 8C, the effect of potential scan rates on the oxidation currents of FA is described.

Since differential pulse voltammetry (DPV) has the advantage of having an increase in sensitivity and better characteristics for analytical applications, DPV technique was performed in order to determine various FA concentrations; a dynamic range between 1.0 × 10^−7^ and 5.0 × 10^−4^ M and a detection limit of 3.4 × 10^−8^ M were found. This protocol has been applied to real samples—FA tablets and urine samples—by using standard addition method. The results for the determination are summarized in Table 8.

Satisfactory recovery and reproducibility values of the experimental were found for FA, as demonstrated by the mean relative standard deviation (RSD%).

## 3. Conclusions

FA has a crucial role in some extremely important biochemical processes including synthesis of nucleic acid, cell division, growth and development of fetuses. For this reason, FA supplements are widely used to prevent and handle folate deficiency in at-risk groups and also to prevent adverse events associated with antifolate medications. Due to the critical rule of FA in human health, during the past years, many efforts have been made in the analytical field in order to develop reliable and precise sensors for its determination.

In more detail, the aim of this short review is to report the most interesting and original electrochemical tools proposed in the past 5 years for FA determination in pharmaceutical preparations, food supplements and other real samples. In conclusion, all the proposed analytical platforms can be applied to the determination of FA in a plethora of real samples: human urine, blood serum, pharmaceutical or commercial preparations. Remarkable recovery values and relative standard deviations have been found in all the proposed research papers. Among all, particularly interesting is the device based on Fe_3_O_4_@SiO_2_/GR nanocomposite modified graphite SPE, which shows good recovery values and RSD% in the simultaneous determination of FA by means of convenient electrochemical sensor. Among traditional sensors, noteworthy is the electrochemical behavior of 2FTNE-modified CMNP paste electrodes (2FTNECMNPPE). Using SWV, the concurrent measurement of epinephrine, uric acid and FA has been realized and no significant differences between the results of the 2FTNECMNPPE and the nominal values of real samples have been observed.

## Figures and Tables

**Table 1:** Summary of analytical methods for the determination of FA in various samples.

Analyte	Technique	LR *	LOD	Sensitivity	RSD%	Ref
FA in pharmaceutical preparations	Spectrophotometric determination by coupling reaction	0.1–8.0 µg mL^−1^	0.0469 µg mL^−1^	0.0066 µg cm^−2^	0.2805	[36]
FA in vegetables	HPLC-UV-Vis	0.3–100 ng mL^−1^	0.1 ng mL^−1^	/	0.3	[39]
FA in beer	LC-MS/MS	/	0.3 µg L^−1^	1.2 µg L^−1^	/	[40]
FA in egg yolks	Modified EMR-lipid method combined with HPLC-MS/MS	0.1–100 ng mL^−1^	18.3 ng mL^−1^	/	3.9 (HC)	[41]
					8.1 (MC)	
					10 (LC)	
FA in commercial preparations	Chemiluminometric procedure	6.0–114 µg mL^−1^	2.0 µg mL^−1^	/	1	[44]
	Fluorimetric procedure	0.02–1.1 µg mL^−1^	0.002 µg mL^−1^		0.7	
FA in human urine	Capillary electrophoresis	0.5–6.0 mg L^−1^	0.30 mg L^−1^	/	0.4–0.7(MT)	[45]
					2.0–3.9 (PA)	
					1.2–1.7 (PH)	
FA in pharmaceutical tablets	Capillary electrophoresis with chemiluminescence determination	5.0 × 10^−8^–10^−5^ M	2.0 × 10^−8^ M	/	1.1 (MT)	[46]
					1.5 (PA)	
					4.9 (PH)	
FA in pharmaceutical preparations	Flow-injection/chemiluminescence determination	2.5 × 10^−5^–3 × 10^−7^ M	2.3 × 10^−8^ M	/	3.5	[48]

* LR (linear range), LOD (limit of detection), RSD (relative standard deviation), HC (high-concentration), MC (medium-concentration), LC (low-concentration), MT (migration time), PA (peak area) and PH (peak height).

**Table 2:** The use of 2FTNEMCNPPE in simultaneous quantification of EP, UA and FA in an EP ampule, an FA tablet and urine samples (n = 5). The amounts of EP and FA in ampoules and tablets were found to be equal to 0.98 mg mL^−1^ and 1.01 mg/tablet, respectively. These outcomes show no significant difference between the results of the 2FTNEMCNPPE and the nominal value on the ampoule label and tablet label (1.00 mg mL^−1^ and 1.00 mg/tablet, respectively) [50].

Sample	Spiked (µM)			Found (µM)			Recovery (%)			R.S.D. (%)		
	EP	UA	FA	EP	UA	EP	EP	UA	FA	EP	UA	FA
EP ampoule	0	0	0	9.0	-	-	-	-	-	2.7	-	-
	2.5	15.0	17.5	11.4	15.5	17.1	99.1	103.3	97.7	3.2	1.9	2.8
	5.0	25.0	27.5	14.3	24.8	27.9	102.1	99.2	101.4	3.1	2.3	2.7
	7.5	35.0	37.5	17.1	35.1	37.3	103.6	100.3	99.5	1.9	3.3	2.4
	10.0	45.0	47.5	18.5	45.6	48.8	97.3	101.3	102.7	2.2	1.8	3.1
FA tablet	0	0	0	-	-	17.0	-	-	-	-	3.4	-
	5.0	17.5	2.5	5.1	17.1	19.7	102.7	97.7	101.0	2.3	1.9	3.2
	10.0	2.5	5.0	9.8	22.9	21.9	98.0	101.8	99.5	3.1	2.3	1.9
	15.0	27.5	7.5	15.1	27.1	24.3	100.7	98.5	99.2	1.7	2.8	2.7
	20.0	32.5	10.0	19.8	33.5	27.5	99.0	103.1	101.8	2.8	3.1	1.8
	0	0	0	-	10	-	-	-	-	-	-	-
	7.5	10.0	30.0	7.4	20.2	30.9	98.7	101.0	103.0	2.9	3.2	1.6
	12.5	20.0	40.0	12.7	29.5	39.1	101.6	98.3	97.7	3.4	2.7	2.6
	17.5	30.0	50.0	18.1	41.2	49.5	103.4	103.0	99.0	1.6	2.6	3.1
	22.5	40.0	60.0	22.4	49.8	61.5	99.5	99.6	102.4	2.2	1.8	2.9

**Table 3:** Real sample analysis of FA using PE/CuO-CNTs/BDHFP (n = 4) [52].

Sample	FA Added (µM)	FA Expected (µM)	FA Found (µM)	Recovery %
Orange Juice	/	/	9.89 ± 0.54	/
	10.00	19.89	20.21 ± 0.87	101.6
Apple Juice	/	/	8.51 ± 0.34	/
	10.00	18.51	18.38 ± 0.65	99.29

**Table 4:** Determination of FA in drug tablet and urine samples by applying differential pulse voltammetry at the surface of CPE [53].

Sample	Initial Found (µM)	Added (µM)	Found (µM)	Recovery (%)
TABLET	3.0	0.0	2.82	94
	3.0	1.0	4.14	104
	3.0	2.0	5.11	102
	3.0	3.0	6.07	101
	3.0	4.0	6.86	98
URINE	0.0	0.0	0.00	-
	0.0	3.0	3.07	102
	0.0	6.0	6.20	103
	0.0	9.0	8.84	98

**Table 5:** Determination of TBHQ and FA in real samples (n = 4) [54]. The different results are compared to those from previously published methods for TBHQ [57] and FA [56], respectively.

Sample	TBHQ Added	FA Added	Found TBHQ Proposed Method	Found TBHQ Published Method	Found FA Proposed Method	Found FA Published Method
Soybean oil	-	-	2.6 ± 0.2	2.6 ± 0.2	-	-
	5.00	-	7.5 ± 0.3	7.7 ± 0.4	-	-
Sesame oil	-	-	5.7 ± 0.4	5.8 ± 0.6	-	-
	10.00	-	15.4 ± 0.5	15.3 ± 0.6	-	-
Apple juice	-		-	-	10.4 ± 0.7	10.2 ± 0.8
	-	10.00	-	-	20.6 ± 0.7	20.1 ± 0.8
Drinking water	10.00	10.00	9.8 ± 0.7	10.5 ± 0.6	10.5 ± 0.5	9.8 ± 0.8

**Table 6:** Summary of biosensors based on SEPs for the determination of FA in various samples.

Analyte	Technique	LOD	Working Range	Sample	Ref.
Vitamin B9 in real specimens.	SPE modified with La+3/Co3O4 nano-cubes.	0.3 µM	1–600 µM	Human urine samples, tablet.	[58]
N-acetylcysteine in the presence of paracetamol and folic acid	CPE modified with Pt-Co nanoparticles and 2-(3,4 dihydroxy phenethyl) isoindoline-1,3-dione	0.04 µM	0.08–650 µM	Human urine samples, tablet.	[59]
Simultaneous determination of sulfisoxazole and folic acid.	CuO Nanoparticles decorated on SWCNT nanocomposite modified CPE.	0.8 µM	0.07–500 µM	Human urine and tablet.	[59]
Folic acid in real specimens.	SPE modified with Graphene Oxide Nanoribbons	0.02 µM	0.1–1600 µM	Human urine samples, tablet	[60]
Folic acid in real specimens.	Mn-zeolite/Graphite modified Screen-printed Carbon Electrode	0.003 µM	0.004–1 µM	Pharmaceutical samples	[61]
Folic acid in real specimens.	SPE modified using GNS-MoS2-AuNPs	38.5 nM	50 nM–1150 µM	Human urine	[62]
Simultaneous determination of folic acid and epinephrine	Graphite SPE modified with Fe3O4@SiO_2_	1 μM	5–1000 μM	Human blood serum and urine	[63]
Folic acid in real specimens.	SPE modified with NiFe_2_O_4_ nanoparticles	0.034 μM	0.1–500 μM	Human urine	[64]

**Table 7:** Determination of epinephrine and FA in human blood serum, urine, epinephrine injection and folic acid tablet samples. All the concentrations are in µM (n = 5) [63].

Sample	Spiked		Found		Recovery, %		Rsd %	
	Epinephrine	FA	Epinephrine	FA	Epinephrine	FA	Epinephrine	FA
Human blood serum	0	0	-	-	-	-	-	-
	10.0	5.0	10.3	4.9	103.0	98.0	3.2	2.4
	20.0	60.0	19.8	61.6	99.0	102.7	17	2.7
Urine	0	0	-	-	-	-	-	-
	12.5	45.0	12.3	45.3	98.4	100.7	2.4	3.1
	22.5	55.0	23.1	53.7	102.6	97.6	1.8	2.8
Epinephrine Injection	0	0	10.5	-	-	-	3.2	-
	2.5	30.0	12.7	30.3	97.7	101.0	1.9	2.6
	5.0	39.7	15.9	40.3	102.6	99.2	2.4	3.3
Folic Acid Tablet	0	0	-	15.0	-	-	-	2.7
	5.0	25.0	4.9	0.9	98.0	102.2	2.4	1.6
	10.0	35.0	10.1	49.2	101.0	98.4	2.7	3.0

**Table 8:** Determination of FA in FA tablet and urine samples. All the concentrations are in µM (n = 5) [64].

Sample	Spiked	Found	Recovery (%)	Rsd (%)
Folic acid tablet	0	15.0	-	3.2
	2.5	17.8	101.7	1.7
	5.0	19.5	97.5	2.8
	7.5	23.3	103.5	2.2
	10.0	24.8	99.2	2.4
Urine	0	-	-	-
	10.0	10.3	103.0	3.4
	20.0	19.9	99.5	1.7
	30.0	29.1	97.0	2.3
	40.0	40.5	101.2	2.8

## Data Availability

Not applicable.
